# A Rare Case of Thyroglossal Duct Cyst Cancer and Literature Review

**DOI:** 10.1210/jcemcr/luad036

**Published:** 2023-04-11

**Authors:** Ashima Mittal, Ariel Sandhu, Mamta Chhetri, Gayatri Jaiswal

**Affiliations:** Department of Endocrinology, Allegheny General Hospital, Pittsburgh, PA 15212, USA; Department of Pathology, Allegheny General Hospital, Pittsburgh, PA 15212, USA; Department of Endocrinology, Allegheny General Hospital, Pittsburgh, PA 15212, USA; Department of Endocrinology, Allegheny General Hospital, Pittsburgh, PA 15212, USA

**Keywords:** thyroglossal duct cyst cancer, thyroid cancer, Sistrunk procedure, thyroglossal duct cyst, papillary thyroid cancer

## Abstract

Thyroglossal duct cyst is the most common thyroid developmental abnormality with a prevalence of 7%, but thyroglossal duct cyst cancer is rare. The incidence of thyroglossal duct cyst cancer is about 1%. The diagnosis is limited by low yield on fine-needle aspiration biopsy (FNAB), and most cases are diagnosed after surgery. There is a paucity of data on the utility of thyroglobulin washout for diagnosis of thyroglossal duct cyst cancer, and it has not been mentioned in previous case reports/series. Papillary thyroid cancer is the most common pathology. Preoperative planning is important as the decision about total thyroidectomy with the Sistrunk procedure (excision of the thyroglossal duct cyst, middle part of hyoid bone, and surrounding tissue around the thyroglossal tract) depends on the presence of clinical or radiological thyroid abnormality. Thyroglossal duct cyst cancer has an excellent prognosis. However, owing to a lack of standard of care for this type of thyroid cancer, there is institutional variability in management. We present a case of thyroglossal duct cyst cancer in a man presenting with painless midline neck swelling. Imaging was suspicious for thyroglossal duct cyst cancer. FNAB was benign. The patient underwent the Sistrunk procedure and pathology was positive for papillary thyroid cancer.

## Introduction

Thyroglossal duct cyst is the most common developmental anomaly of thyroid, with a prevalence of 7% [1]. Thyroglossal duct cyst cancer, however, occurs in approximately 1% of thyroglossal duct cysts [2]. Given its rarity, it is important for physicians to be familiar with its clinical features, surgical management and prognosis. We present a rare case of thyroglossal duct cyst cancer in a young man presenting with a painless midline neck swelling.

## Case Presentation

A 37-year-old man with a past medical history of hypertension and hyperlipidemia presented with a painless midline neck swelling. He noted the swelling after cleaning his long-time beard. He did not report complaints of difficulty in swallowing, breathing, or voice changes. Family history was negative for thyroid cancer.

## Diagnostic Assessment

**Figure 1.**
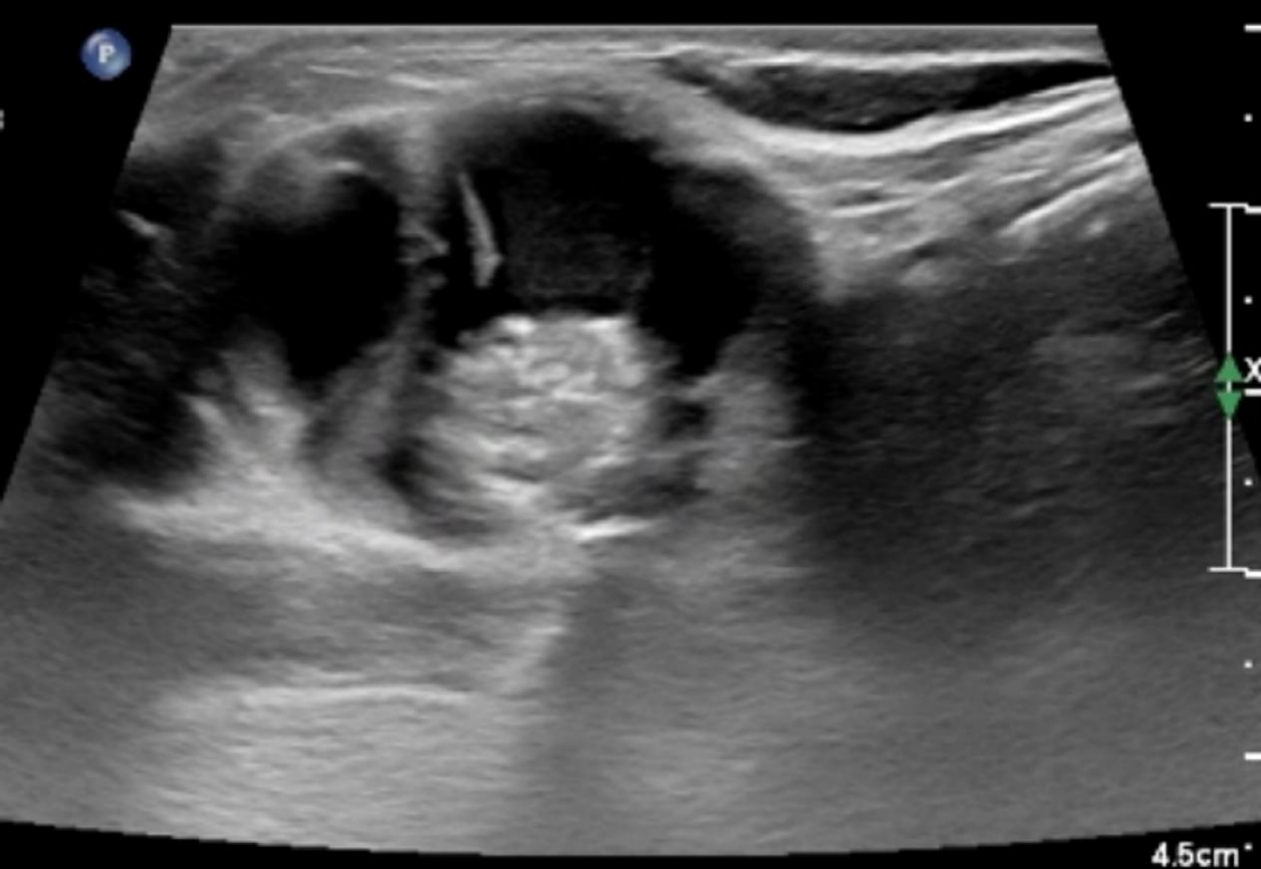
Ultrasound image showing complex solid cystic mass with visible septations.

**Figure 2.**
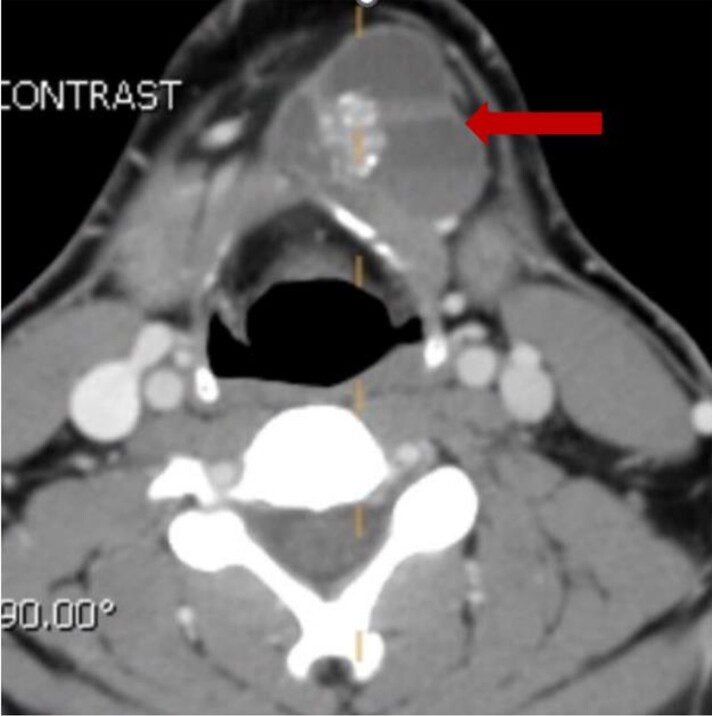
Computed tomography image—axial section showing multiloculated infra hyoid cystic mass with internal punctate calcifications and fluid-fluid levels.

Thyroid function tests showed free thyroxine of 1.02 ng/dL (0.82-1.77 ng/dL) (13.15 pmol/L) and thyrotropin of 1.910 μIU/mL (0.450-4.50 μIU/mL) (1.910 mIU/L). Thyroid ultrasound showed a complex mixed solid and cystic mass in the midline measuring 4.07 × 2.42 × 4.01 cm with a posterior solid 1.4-cm nodular component with increased vascularity (Fig. 1). No thyroid nodules were noted in the thyroid lobes. No suspicious lymph nodes were noted. Fine-needle aspiration biopsy (FNAB) of the anterior neck mass was benign. Computed tomography (CT) of the neck soft tissue showed a multiloculated left neck infrahyoid cystic mass with thick internal septations, soft tissue component, and punctate calcifications and fluid-fluid levels, findings concerning for thyroglossal duct cyst carcinoma (Fig. 2).

## Treatment

**Figure 3.**
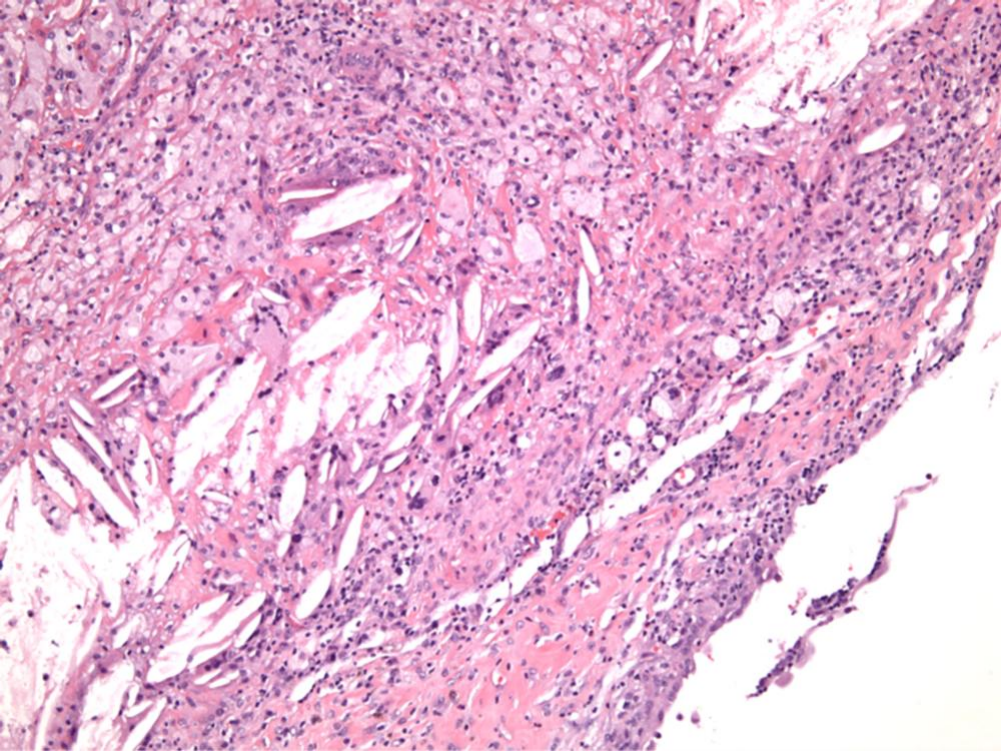
Hematoxylin and eosin, 100×: The tissue predominantly showed degenerated thyroglossal duct cyst wall with denuded epithelium, abundant foamy macrophages, and cholesterol clefts.

**Figure 4.**
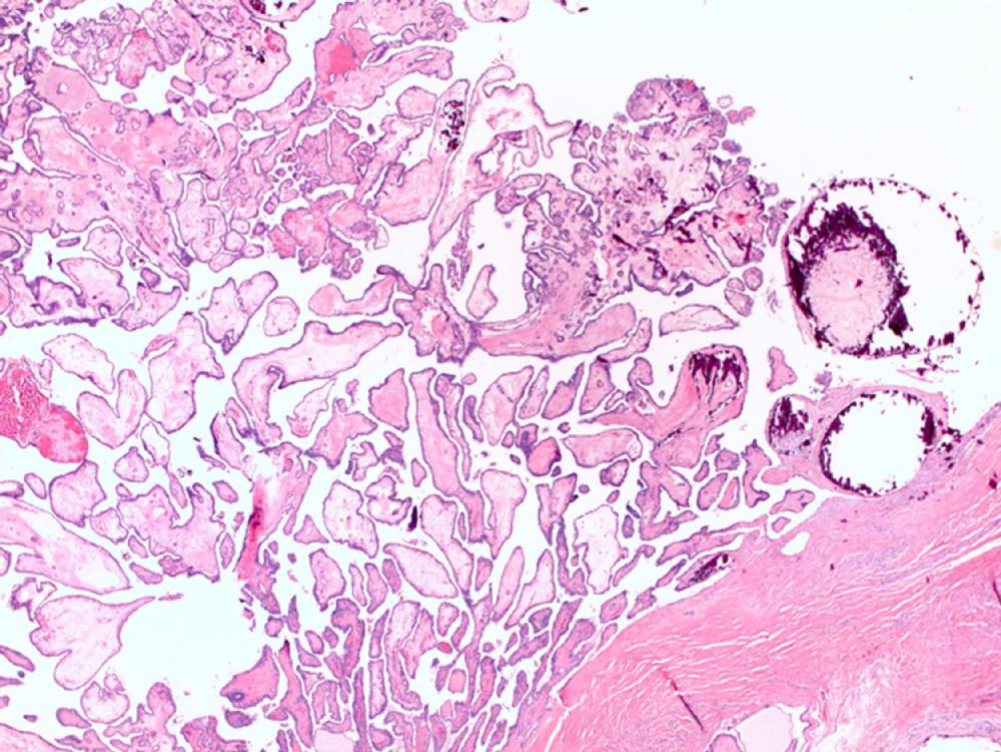
Hematoxylin and eosin, 20×: The tumor showed complex arboreal papillary architecture with hyalinized fibrovascular cores. Psammomatous calcifications involve the tips of many papillae.

**Figure 5.**
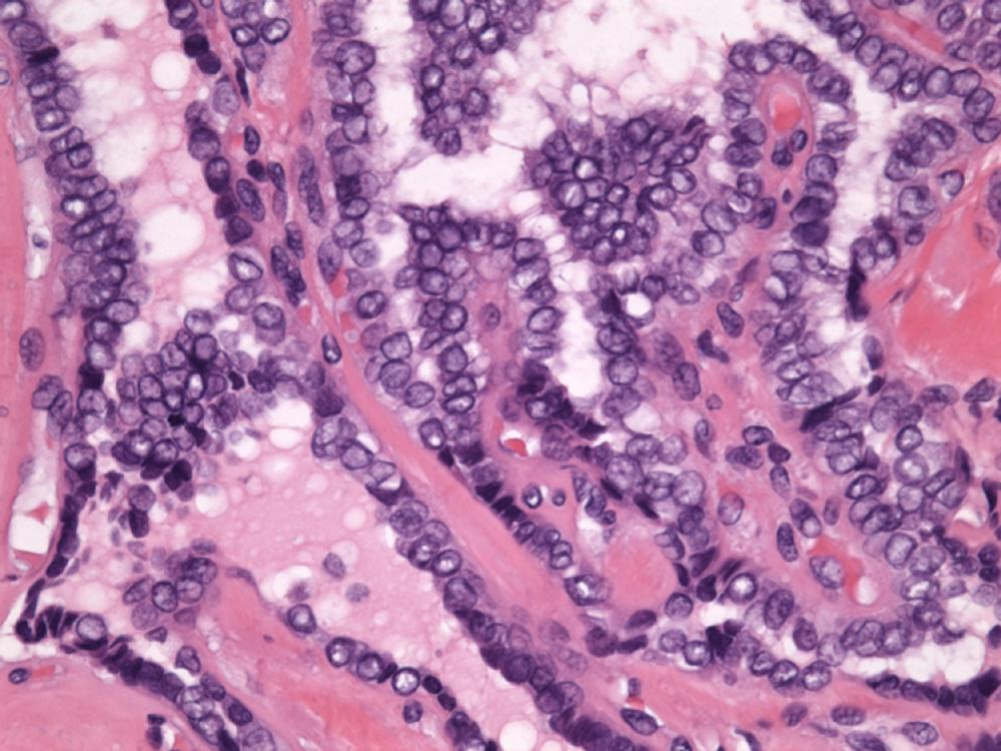
Hematoxylin and eosin, 400×: Cytologic features of papillary thyroid cancer are seen including nuclear grooves, nuclear enlargement, nuclear overlapping, and high nuclear-to-cytoplasmic ratios. The marginated chromatin pattern and optically clear nuclei demonstrate the classic “Orphan Annie Eye” appearance.

**Figure 6.**
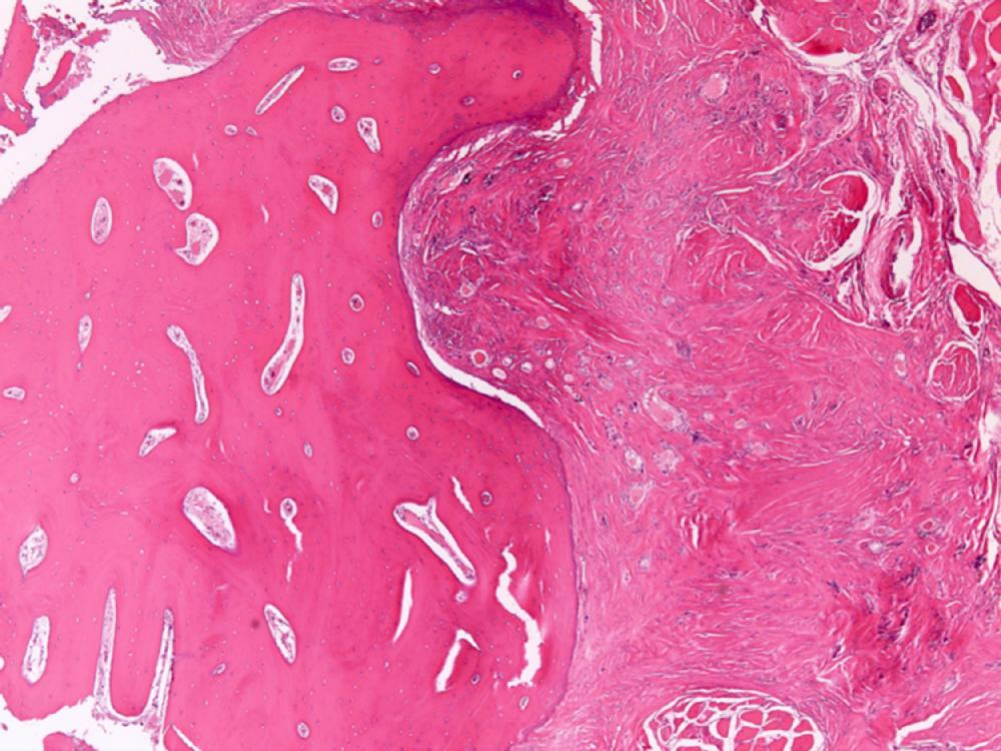
Hematoxylin and eosin, 40×: Infiltrative-appearing ectopic thyroid glands are embedded in the fibrous tissue adjacent to the hyoid bone.

Our patient underwent the Sistrunk procedure (excision of the thyroglossal duct cyst, middle part of the hyoid bone and surrounding tissue around the thyroglossal tract). Surgical pathology showed a 0.9-cm papillary thyroid carcinoma arising from a thyroglossal duct cyst with capsular invasion and focal extension into adjacent soft tissue (Figs. 3-5). There was no lymphovascular invasion or involvement of adipose tissue, large nerves, perivascular tissue, skeletal muscle, or bone. A small focus of ectopic thyroid tissue was present adjacent to the hyoid bone (Fig. 6). The pathologic stage was pT1aNx. Immunohistochemistry was positive for CK 17, TTF-1, pan cytokeratin, and PAX-8.

## Outcome and Follow-up

Two months postoperatively, laboratory workup showed thyroglobulin of 6.3 ng/mL (radioimmune assay) (3-40 ng/mL) (6.3 μg/L) and thyroglobulin antibody of 1.6 IU/mL (< 1 IU/mL) (1.6 kIU/L), which is normal for presence of thyroid gland. Postoperative thyroid ultrasound did not show evidence of thyroid nodules. Thyroid ultrasound and CT soft tissue neck at 1 year (last follow-up) were negative for thyroid nodules or lymph nodes. Given the small focus of thyroid cancer with low-risk surgical pathology on resection and ultrasound findings of a normal thyroid gland, the plan is to monitor the patient annually without additional thyroidectomy.

## Discussion

Thyroglossal duct cyst develops from persistent thyroglossal duct, which connects the thyroid gland to the base of the tongue. It is the most common cervical congenital abnormality, with a prevalence of 7% [1]. Thyroglossal duct cyst cancer affects 1% of these thyroglossal duct cysts [2]. Most evidence favors thyroglossal duct cyst cancers arising de novo from a thyroglossal duct cyst, rather than being a metastasis from the thyroid gland [2]. Ectopic thyroid tissue has also been identified in the walls of thyroglossal duct cyst cancers, supporting de novo origin [2]. This is further reinforced by the finding that ectopic tissue does not contain parafollicular C cells, and a lack of case reports of medullary thyroid cancer have been reported in thyroglossal duct cyst. Co-occurrence of papillary thyroid cancer in the thyroid gland along with thyroglossal duct cyst cancer are therefore thought to be multifocal cancers, rather than metastasis [2].

The most common presentation is painless midline neck mass with or without compressive symptoms. It is more common in women than man [3]. Median age of presentation is in the third to fourth decade [2, 3]. Imaging studies like ultrasound scan and CT of the neck are important preoperatively as these could provide diagnostic clues. Mural mass with calcifications may suggest thyroglossal duct cyst cancer [4].

In our patient, even though FNAB did not indicate malignancy, the imaging was concerning. Ultrasound and neck CT showed a multiloculated left neck infrahyoid cystic mass with thick internal septations, a soft tissue component, and punctate calcifications and fluid-fluid levels. Given the imaging characteristics along with reaccumulation of cyst fluid post aspiration, there was high suspicion for malignancy, which led to the decision to pursue surgery. Thyroglossal duct cyst cancer is an uncommon preoperative diagnosis, owing to the low true positive rate of FNAB. It is mostly a histopathological diagnosis postoperatively. The diagnostic rates of FNAB have been variable [5]. This has been attributed to low cellularity of the sample due to cystic contents [5]. Optimal FNAB yield is achieved by carrying out a biopsy of the solid component of the cyst or repeating the biopsy after aspiration of cystic contents. Thyroglobulin washout can be performed if there is a high index of suspicion for thyroglossal duct cyst cancer. There is a paucity of data on the utility of thyroglobulin washout in diagnosis of thyroglossal duct cyst cancer, and it has not been mentioned in previous case reports/series. Papillary thyroid cancer is the most common histological diagnosis followed by other mixed papillary/follicular thyroid cancer and squamous cell cancer [2]. Rare cancer types including anaplastic cell cancer and Hürthle cell cancer have been reported [2]. In comparison to intrathyroidal papillary thyroid cancer, thyroglossal duct cyst papillary thyroid cancer has a higher rate of skip metastasis, that is, the tendency to involve lateral compartment lymph nodes without central compartment involvement [6]. This is due to thyroglossal duct cyst anatomy and lymphatic drainage. Both entities are similar in terms of multifocality and prognosis [6].

The lymph node bed is the most common site of recurrence with a mean time to recurrence of 42.1 months and a recurrence rate of 4.3% [3]. Thyroglossal duct cyst cancer has an excellent prognosis, with a 5-year survival of more than 90% [7]. Less common histological types like squamous cell thyroglossal duct cyst cancer present at more advance stages and are associated with worse prognosis [2].

Treatment options include the Sistrunk procedure in all cases with or without total thyroidectomy/lateral and central neck dissection and radioiodine therapy. There is a difference of opinion in the literature with regard to total thyroidectomy with the Sistrunk procedure for routine treatment of thyroglossal duct cyst cancer. The high incidence of coexistence of thyroid cancer with thyroglossal duct cyst cancer (25%-56%) and better postoperative monitoring favor empirical total thyroidectomy [8, 9]. Low-risk features like tumor size less than 1 cm, negative margins, and the absence of distant metastasis favor against routine total thyroidectomy [10]. Other considerations to include total thyroidectomy and I-131 therapy routinely include older patients (age > 45 years), prior history of head and neck radiation, concomitant thyroid cancer, high risk histological features like tumor size greater than 4 cm, soft tissue extension, and distant metastasis with clinically or radiologically abnormal lymph nodes [7, 10].

Our decision to pursue a Sistrunk procedure without total thyroidectomy in this case was based on multiple considerations: the patient’s age, absence of suspicious lymph nodes or thyroid nodules, low-risk surgical pathology, and overall excellent prognosis. We plan to closely monitor the patient for any signs of malignancy.

There is no indication for postoperative levothyroxine therapy in patients treated with Sistrunk procedure only compared to those treated with additional total thyroidectomy [2]. Goal thyrotropin should be based on risk stratification of surgical pathology per American Thyroid Association guidelines. These treatment guidelines have not been addressed in American Thyroid Association guidelines given the rarity of thyroglossal duct cyst cancer.

Postoperatively, periodic monitoring with neck CT and/or ultrasound is important in patients treated with the Sistrunk procedure only, given the high incidence of synchronous malignancy in the thyroid gland. To conclude, there should be a low threshold for FNAB/total thyroidectomy in case of thyroid abnormalities detected clinically or on imaging.

## Learning Points

- Thyroglossal duct cyst cancer is a rare cancer with excellent prognosis in most reported cases.
- It is an uncommon preoperative diagnosis, owing to the low true positive rate of FNA biopsy.
- Postoperatively, periodic monitoring with neck CT and/or ultrasound is important in patients treated with the Sistrunk procedure only, given the high incidence of synchronous malignancy in the thyroid gland.
- It is imperative to know about this entity because surgical planning depends on the detection of thyroid abnormalities clinically or on imaging as total thyroidectomy would be required in addition to a Sistrunk procedure in that case.
- Intrathyroidal papillary thyroid cancer and thyroglossal duct cyst papillary thyroid cancer are similar in terms of multifocality and prognosis.
- Skip metastasis is more common in thyroglossal duct cyst papillary thyroid cancer compared to intrathyroidal papillary thyroid cancer.

## Contributors

A.M. and G.J. composed the manuscript and literature review. A.S. provided the figures and conducted the pathology review. A.M., G.J., and M.C. were responsible for the acquisition, analysis, or interpretation of data for the work, revising it critically for important intellectual content, and gave final approval of the version to be published. All authors contributed to the article and approved the submitted version.

## Data Availability

Original data generated and analyzed during this study are included in this published article.

## Abbreviations

CT: computed tomography
FNAB: fine-needle aspiration biopsy
